# Surgically induced necrotizing scleritis after orbital surgery with intraconal tumor excision: a case report

**DOI:** 10.1007/s10792-026-04019-5

**Published:** 2026-03-03

**Authors:** Sylves Patrick, Jasmine Rashid, Shee Wen Chua, Amalina Juares Rizal, Hanida Hanafi

**Affiliations:** 1 Department of Ophthalmology, Faculty of Medicine and Health Sciences, Universiti Malaysia Sabah (UMS), 88400 Kota Kinabalu, Sabah Malaysia; 2 Oculoplastic and Reconstructive Unit, Department of Ophthalmology, Queen Elizabeth Hospital, 88586 Kota Kinabalu, Sabah Malaysia

**Keywords:** SINS, Necrotizing scleritis, Surgically induced necrotizing scleritis, Orbitotomy, Orbital surgery

## Abstract

**Background:**

Surgically induced necrotizing scleritis (SINS) is a blinding ocular disease characterized byinfl ammation with scleral necrosis. It occurs as early as one day after ocular surgery, although it can also occur years later. Commonly, it occurs after pterygium and cataract surgeries

**Case Presentation:**

Here, we report a case of a 40-year-old woman with underlying diabetes mellitus whounderwent a successful excisional biopsy via a swinging upper eyelid approach with lateral canthotomy andcantholysis, along with disinsertion of the superior and lateral rectus muscles, for an orbital cavernous venousmalformation. However, 9 weeks postoperatively, the patient developed persistent pain in the right eye (RE),especially with lateral gaze, which was not relieved with oral ibuprofen. At 16 weeks postoperative, an RE focal areaof scleral necrosis with surrounding conjunctival injection was noted at the superotemporal bulbar region. Magneticresonance imaging of the orbit and brain revealed RE focal thickening at the posterolateral sclera, posterior to thelateral rectus muscle insertion, suggesting posterior scleritis. The patient was treated with oral prednisolone (40mg/day) followed by a tapering regimen with adjunctive methotrexate (20 mg/week). After three months of oralprednisolone and six months of methotrexate, her disease resolved with no complications.

**Conclusion:**

SINS is a rare but potentially sight-threatening complication following orbital surgery with intraconaltumor excision. Persistent pain can be a useful clue before the clinical signs become apparent.

## Introduction

Scleritis is the inflammation of the sclera. It can be caused by infection or autoimmune disease, and it can be surgically induced. Surgically induced necrotizing scleritis (SINS) is rare and occurs after ocular surgery [1]. It is characterized by scleral necrosis at or near the surgical site or, more rarely, distant from the surgical site [2]. SINS has been reported to occur after pterygium surgery, strabismus surgery, vitreoretinal surgery, trabeculectomy, or cataract surgery [2–6]. However, SINS mainly occurs after pterygium surgery or cataract surgery [6, 7]. SINS can progress very fast and cause corneal or scleral perforation with irreversible blindness. Here, we report a case of SINS that occurred after orbital surgery, as well as its outcomes.

## Case report

**Fig. 1:**
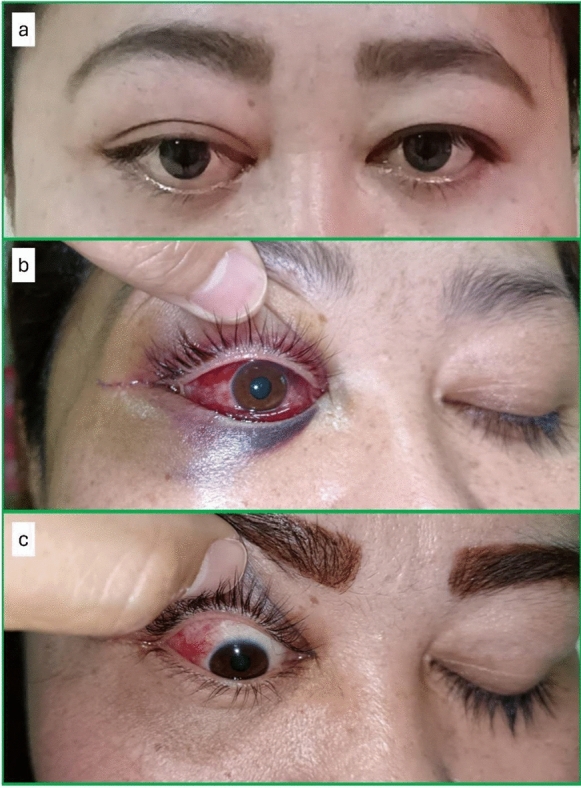
Pre- and postoperative findings **a** RE axial proptosis; **b** RE subconjunctival hemorrhage with periorbital hematoma at postoperative day 2; **c** RE redness improving at postoperative 8 weeks

A 40-year-old woman with underlying diabetes mellitus presented with painless right eye (RE) swelling for three months associated with reduced vision. Examination of the RE revealed axial proptosis, limited ocular motility, and positive relative afferent pupillary defect, while a fundus examination showed choroidal folds (Fig. 1a). The best corrected visual acuity (BCVA) was 6/15 and 6/6 in the RE and left eye (LE), respectively. The LE anterior segment and posterior segment examinations were unremarkable. The systemic examination was also unremarkable.

**Fig. 2:**
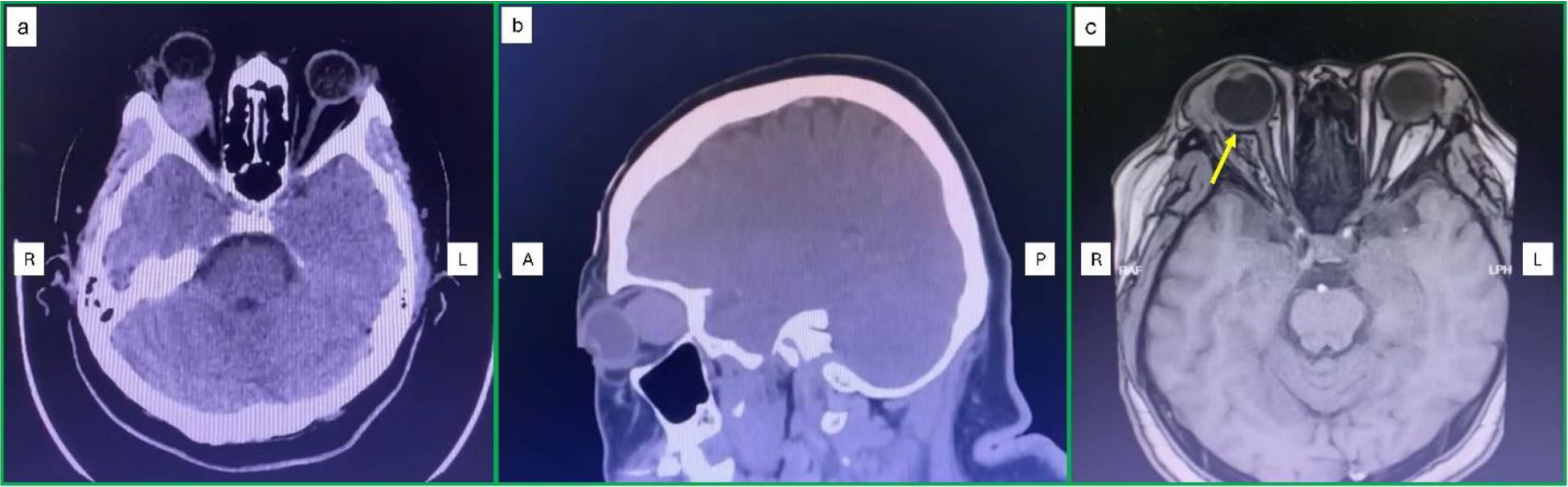
CECT and MRI findings **a**–**b** CECT of the orbit and brain revealed a right orbital well-defined, intraconal mass measuring 2.3 × 2.2 × 2.1 cm, with poor fat plane demarcation from the globe, optic nerve, and lateral rectus muscle; **c** MRI of the orbit and brain showed RE focal thickening at the posterolateral sclera, posterior to the lateral rectus muscle insertion, suggesting posterior scleritis (yellow arrow). R—right, L—left, A—anterior, and P—posterior

Contrast-enhanced computed tomography (CECT) of the orbit and brain revealed a right superolateral orbital, well-defined, heterogeneous, minimally enhancing intraconal mass measuring 2.3 × 2.2 × 2.1 cm, located lateral to the optic nerve and abutting the superior rectus and superior oblique muscles, with poor fat plane demarcation from the globe, optic nerve, and lateral rectus muscle (Fig. 2a, b). The patient underwent excisional biopsy via a swinging upper eyelid approach, consisting of a fornix-based transconjunctival incision from lateral to medial with lateral canthotomy and cantholysis. The lateral portion of the upper eyelid was subsequently mobilized superiorly to expose the superolateral aspect of the orbit. Disinsertion of the superior and lateral rectus muscles was performed to facilitate adequate exposure, without prior muscle retraction. The mass was removed en bloc without the use of a cryo probe, and the extraocular muscles were reinserted using 6–0 Vicryl sutures. The operation was successful and without complications. The patient was discharged well and completed one week of oral cefuroxime 500 mg twice daily. The histopathological examination findings confirmed an orbital cavernous venous malformation, previously known as an orbital cavernous hemangioma. The postoperative findings on day two and week eight are shown in Fig. 1b, c.

**Fig. 3:**
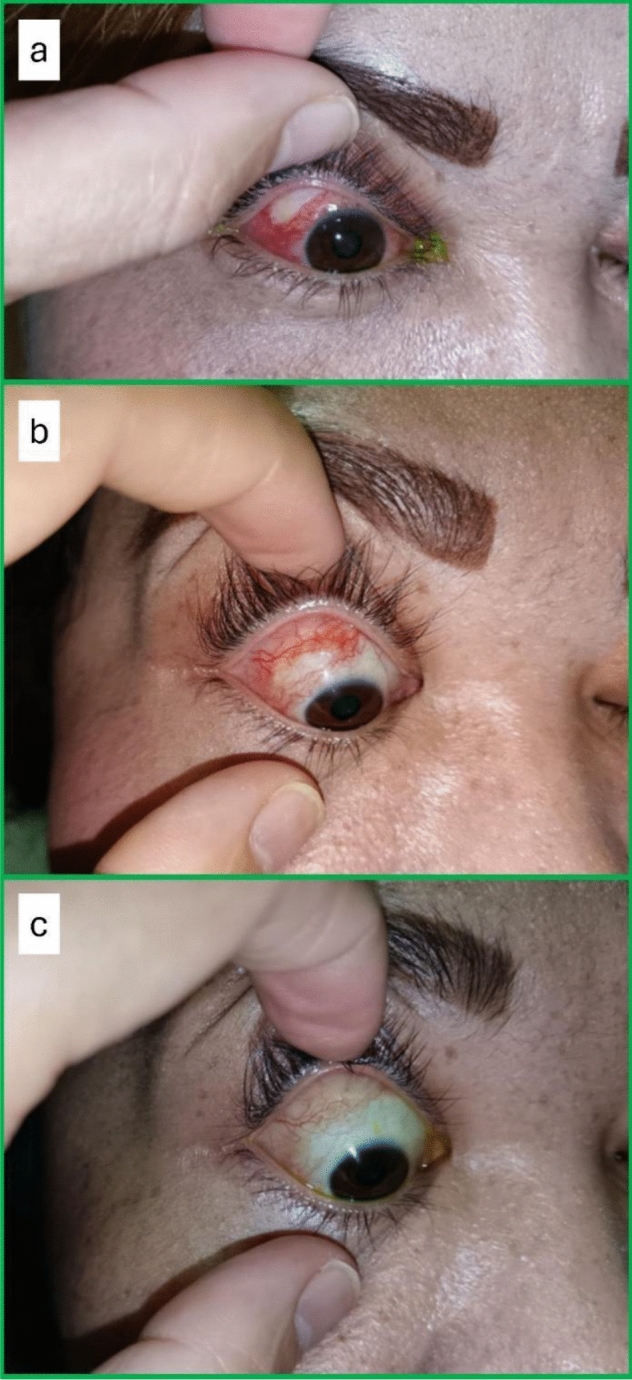
Pre- and post-treatment findings **a** RE focal scleral necrosis at the superotemporal bulbar region with surrounding generalized conjunctival injection at 16 weeks after surgery; **b** At post-treatment 7 weeks, RE focal scleral necrosis showed increased vascularization and decreased in size; **c** At 18 weeks post-treatment, RE focal scleral necrosis had resolved with mild conjunctival redness

However, nine weeks postoperatively, the patient started to have pain in the RE associated with redness. This condition persisted and worsened, even after taking ibuprofen tablets. The pain was aggravated by eye movement, especially with lateral gaze. At 16 weeks post-surgery, an RE focal scleral necrosis with tenderness was noted at the superotemporal bulbar region, with surrounding generalized conjunctival injection (Fig. 3a). Otherwise, the RE visual acuity was 6/15, and the fundus examination was normal. In view of the suspected diagnosis of surgically induced necrotizing scleritis, a comprehensive systemic evaluation was performed to exclude underlying autoimmune, infective, and metabolic causes. The complete blood count, renal function test, liver function test, C-reactive protein, erythrocyte sedimentation rate, thyroid function test, uric acid, antinuclear antibody, rheumatoid factor, antineutrophil cytoplasmic antibodies, complement fractions, chest x-ray, Mantoux test, HIV test, venereal disease research laboratory test, and hepatitis B and C tests were unremarkable.

Contrast-enhanced magnetic resonance imaging (MRI) of the orbit and brain showed RE focal thickening at the posterolateral sclera, posterior to the lateral rectus muscle insertion, suggesting posterior scleritis (Fig. 2c). The patient was started on oral prednisolone at 40 mg once daily for one week, followed by a tapering regimen over a total period of 12 weeks. The dose was reduced to 35 mg, 30 mg, and 25 mg once daily, each for one week, followed by 20 mg, 15 mg, 10 mg, and 5 mg once daily, each for two weeks, before treatment was discontinued. In view of her being unable to tolerate oral prednisolone well due to dyspepsia and the uncontrolled blood sugar level, oral methotrexate 20 mg per week was also started. Dyspepsia was managed with oral omeprazole 20 mg daily, and her oral metformin dosage was adjusted to optimize blood glucose control. Oral folic acid 5 mg once daily, except on the methotrexate dosing day, was prescribed concurrently with methotrexate. The ocular findings after seven weeks and 18 weeks of treatment are shown in Fig. 3b, c. The patient received three months of oral prednisolone and six months of oral methotrexate. After completion of treatment, the eye pain and redness resolved with the same vision and full extraocular muscle movement. At approximately 10 months after completion of treatment no recurrence was observed.

## Discussion

The SINS mechanism is still not fully understood. It is believed that surgical trauma and temporary ischemia alter and expose the tissue antigens at the surgical site to the immune system. Therefore, having never been exposed to the immune system, these antigens are recognized as foreign and trigger a delayed type of hypersensitivity response, leading to necrotizing scleritis [6]. Local ischemia is a known complication of strabismus surgery, which may contribute to the development of SINS following strabismus surgery [8]. The disinsertion of the extraocular muscles likely contributed to the scleral ischemia in our case.

In addition, orbital surgery is related to more surgical trauma in comparison to ocular surgery, especially involving the intraconal space, where the blood supply to the sclera may be compromised during surgery. We believe that, in our case, the surgical trauma and temporary ocular ischemia during globe manipulation also contributed to SINS. To the best of our knowledge, this is the first reported case of SINS following orbital surgery with intraconal tumor excision performed in a single procedure. O’Donoghue et al. reported a case of SINS that occurred after strabismus surgery, preceded by orbital decompression. However, this involved multiple surgeries and no further case details were available [6].

Reportedly, SINS can be associated with many systemic diseases such as collagen vascular disease, thyroid disease, diabetes mellitus, and hyperuricemia. However, the most significant association is with collagen vascular diseases such as rheumatoid arthritis, Wegener's granulomatosis, polyarteritis nodosa, seronegative polyarthropathy, and polymyalgia rheumatica [6]. The only associated risk factor in our case was diabetes mellitus. Optimizing the associated systemic illness is crucial in increasing the likelihood of successful treatment.

A diagnosis of SINS is not always straightforward because the signs may only become apparent later, after the symptoms have occurred. The occurrence of SINS after ocular surgery has been reported to happen as early as one day after surgery, although it has been known to occur up to 51 years later [6, 9]. We believe that, in our case, SINS began after nine weeks, following the period of pain-free recovery after the post-orbital surgery. Persistent pain, redness, and pain that worsened with eye movement were the most critical symptoms at the beginning of our case because the signs of scleral necrosis were only clinically apparent after seven weeks of the onset of pain. In our case, the SINS involved not only the anterior sclera but also the posterior sclera, as evidenced by an MRI scan. Imaging is crucial in ruling out other causes and determining the extent of the disease.

Infectious causes need to be excluded because the treatment plan will be different. SINS can happen with bacterial superinfection, where the generous use of antibiotics is mandatory [10]. Besides that, Sahu et al. reported a case of fungal scleritis masquerading as SINS [11]. Thus, microbiological tests are essential to rule out infectious causes. The use of corticosteroids can worsen the condition of infectious scleritis [11]. Although wound swabs for culture and scleral biopsy were not performed in our case, the significant improvement after administering oral prednisolone and methotrexate suggested a non-infectious cause.

The mainstay of treatment for SINS is systemic corticosteroids with or without immunosuppressive agents [6]. In cases where there is a poor response to steroids, the addition of immunosuppressive agents, such as methotrexate, mycophenolate mofetil, azathioprine, and cyclophosphamide, has been reported as successful in treating SINS [4, 6, 12]. The use of non-steroidal anti-inflammatory agents (NSAIDs) is less effective in treating SINS, as in our case [6]. In more severe SINS, surgical excision of necrotic tissue with tectonic reconstruction using a graft (scleral, corneal, or conjunctival) is required [6, 13]. As the disease can progress very fast, treatment should be started as soon as possible to prevent further progression and complications, such as scleral perforation, corneal perforation, uveal tissue prolapse, phthisis bulbi, and blindness [6, 14].

## Conclusion

SINS is a rare but potentially sight-threatening complication following orbital surgery with intraconal tumor excision. Persistent pain can be a useful clue before the clinical signs become apparent. In this case, multiple factors likely contributed to the development of surgically induced necrotizing scleritis, including extraocular muscle disinsertion, underlying diabetes mellitus, and orbital surgery itself. While muscle disinsertion and diabetes may have played significant roles, other contributing factors cannot be completely excluded. A high index of suspicion is paramount and must be included in the differential diagnoses. Careful examination and appropriate investigations are crucial so that treatment can be started without undue delay in order to prevent visual loss.

## Data Availability

No datasets were generated or analysed during the current study.
